# Novel autonomic dysregulation score in children with hypothalamic syndrome

**DOI:** 10.1530/EC-25-0336

**Published:** 2025-12-01

**Authors:** Nathalie J Doelman-Oldenburger, Hermann L Müller, Hanneke M van Santen

**Affiliations:** ^1^ Princess Máxima Center, Utrecht, The Netherlands; ^2^ Department of Pediatric Endocrinology, Wilhelmina Children’s Hospital, University Medical Center, Utrecht, The Netherlands; ^3^ University Children’s Hospital, Carl von Ossietzky Universität Oldenburg, Department of Pediatrics and Pediatric Hematology/Oncology, Klinikum Oldenburg AöR, Oldenburg, Germany

**Keywords:** hypothalamic syndrome, ROHHAD, diagnostic criteria, hypothalamic obesity

## Abstract

**Objective:**

Rapid-onset obesity with hypothalamic dysfunction, hypoventilation and autonomic dysregulation (ROHHAD) is a rare syndrome manifesting in childhood. Diagnosis of ROHHAD syndrome remains challenging due to the diversity of symptoms that may be missed easily, especially for autonomic dysfunction, and partly develop later in time. The 2023 hypothalamic syndrome (HS) diagnostic criteria are a novel tool for recognizing symptoms of hypothalamic dysfunction. However, symptoms of autonomic dysregulation are lacking in these criteria. They are therefore insufficient for use in ROHHAD patients. No other scoring system for autonomic dysfunction in ROHHAD syndrome exists. We aim to improve the diagnostic criteria for HS by including a score for autonomic dysregulation, supporting early diagnosis of ROHHAD syndrome.

**Methods:**

A score for autonomic dysregulation in ROHHAD syndrome supplementary to the 2023 HS diagnostic criteria was developed based on existing instruments to assess autonomic dysfunction symptoms adjusted for specific symptoms in ROHHAD syndrome, with a score ranging from 0 to 10. The diagnostic criteria for HS including our add-on were tested retrospectively in four ROHHAD patients.

**Results:**

Four ROHHAD patients, median age 9.4 years (range 4.6–25.7), were assessed regarding signs and symptoms of HS and autonomic dysfunction. All patients had HS and scored on at least three different domains of autonomic dysregulation. Median score was 9 out of 10 (range 4–9).

**Conclusions:**

The 2023 HS diagnostic criteria are not sufficient to recognize autonomic dysfunction due to hypothalamic dysfunction in ROHHAD syndrome. Our add-on score is clinically easy to use and may help in early recognition and follow-up in ROHHAD syndrome and other causes of HS.

**Significance statement:**

Rapid-onset obesity with hypothalamic dysfunction, hypoventilation and autonomic dysregulation (ROHHAD) is a rare cause of HS in childhood. In the recently developed diagnostic criteria for HS, the autonomic manifestations of hypothalamic dysfunction, which are common in ROHHAD syndrome, are not represented sufficiently. Children with ROHHAD may, for example, experience ophthalmological problems (strabismus or oculomotor apraxia), altered pain threshold, increased or impaired sweating, cold extremities, and/or heart rate dysregulation. Many of these symptoms are mostly unnoticed by the clinician or caregivers, leading to a delay in diagnosis and treatment. An add-on to the diagnostic criteria for HS including parameters of autonomic dysregulation will support early recognition of ROHHAD syndrome, follow-up during treatment and research purposes.

## Introduction

The hypothalamic syndrome (HS) is a rare but clinically severe condition (1). The hypothalamus is responsible for hunger and satiety regulation, sleep and circadian rhythm, temperature regulation, behavior and regulates the pituitary gland. Dysfunction of the hypothalamus can therefore affect many different clinical domains (2, 3). It can lead to hyperphagia, obesity, sleep disorders, hypo- or hyperthermia, hypopituitarism and behavioral issues. In addition, it plays a major role in regulation of the autonomic nervous system. Stimulation of the anterolateral hypothalamic region promotes parasympathetic response, whereas stimulating the posteromedial region results in sympathetic activation. Normally, the hypothalamus keeps a balance between the autonomic functions of these two regions. Lesions or dysfunction of the respective regions may result in an opposite or dysfunctional response, with stimulation of the sympathetic nervous system by the anterolateral region and/or stimulation of the parasympathetic nervous system by the posteromedial region (4). Subsequently, both sympathetic and parasympathetic activation can be encountered in patients with hypothalamic dysfunction. Symptoms of autonomic dysfunction can range from ophthalmological abnormalities such as strabismus or decreased tearing, altered sweating, increased or decreased sensitivity to pain, to orthostatic hypotension or cardiac arrhythmias (5).

There are multiple causes for HS in childhood. It may be due to a genetic cause, such as in the Prader–Willi syndrome or Bardet–Biedl syndrome, or of acquired origin, such as caused by (treatment for) a brain tumor in the suprasellar region, of which craniopharyngioma, low-grade glioma or germ cell tumors are most frequently seen (1). Further causes are traumatic brain injury (TBI), septo-optic dysplasia (SOD), hypophysitis/hypothalamitis and hypothalamic hamartoma (6, 7, 8, 9, 10). Another cause of HS, possibly of autoimmune or paraneoplastic etiology is the rapid-onset obesity with hypothalamic dysfunction, hypoventilation and autonomic dysregulation (ROHHAD) syndrome (5). All these different causes of hypothalamic dysfunction are very rare and can present with a different range of symptoms. The different clinical manifestations of hypothalamic dysfunction in brain tumors have recently been described by our group (11). Within a group of 106 pediatric patients diagnosed with HS after treatment for a suprasellar brain tumor (40 craniopharyngioma, 61 low-grade glioma and 5 germ cell tumors), 42 different clinical presentations of hypothalamic dysfunction could be distinguished. It was shown that HS does not always present with obesity and pituitary dysfunction but can present in various ways. One of the faces that was not described is the typical ‘face’ of HS that is seen in children with ROHHAD syndrome, yet children with ROHHAD have another ‘different face’ of HS.

ROHHAD syndrome is a rare, progressive pediatric syndrome with around 200 cases reported in the literature. A neural crest tumor is present in around 50% of patients with ROHHAD syndrome; therefore, it is also known as ROHHAD-NET syndrome. It is characterized by rapid-onset obesity and hypothalamic dysfunction in a previously healthy young child (12). Typically, central hypoventilation and autonomic dysfunction develop later in time, although children can already show symptoms of autonomic dysregulation at first presentation (13). This varying presentation makes the diagnosis of ROHHAD syndrome very challenging and often results in a delay of diagnosis. Early recognition of ROHHAD and HS in general is critical, however, not only for treatment of hypothalamic dysfunction but also for the other possible consequences of underlying etiology (such as visual impairment in case of a brain tumor). In children with ROHHAD syndrome, delay in diagnosis might lead to unrecognized cardiorespiratory complications and even cardiorespiratory arrest. There is a significant risk of cardiorespiratory arrest in ROHHAD syndrome reported of up to 60%, although no clear data on mortality or long-term outcome are available. There is currently no cure available and multidisciplinary management is largely supportive. Some case reports have described treatment with immunotherapy to target a possible underlying autoimmune etiology, with variable effect. Agents that have been tried include cyclophosphamide, rituximab, IV immunoglobulins and various steroid preparations (14). For obesity management, some cases have also shown benefit with GLP-1 agonists (14).

For these reasons, clinical criteria for early recognition and diagnosis of HS are essential and have recently been proposed (3). However, the signs and symptoms of autonomic dysregulation typical for hypothalamic dysfunction in ROHHAD syndrome are not sufficiently included in this score yet. In fact, no scoring system for autonomic symptoms in hypothalamic dysfunction currently exists. There are validated self-assessment instruments of autonomic function designed for a broad range of autonomic dysfunction symptoms, for example the Composite Autonomic Symptom Score (COMPASS) 31 or the Survey of Autonomic Symptoms (SAS), which is designed for dysautonomia in patients with diabetic neuropathy (14, 15). The Pediatric Autonomic Symptoms Scale (PASS) is focused on familial dysautonomia and autism spectrum disorders and consists of 80 questions (16). The COMPASS-31, the instrument used most frequently in different causes of autonomic dysregulation, is lacking questions on altered pain threshold, ophthalmological dysfunction and cardiovascular manifestations compared to the symptoms previously reported in ROHHAD syndrome (5), whereas problems with gastroparesis are not typically seen in ROHHAD patients. Both the PASS and COMPASS questionnaires are very specific for particular disorders and too broad for children with hypothalamic dysfunction. In addition, both questionnaires are quite long, which may hamper its use, especially taking into account all other domains of hypothalamic dysfunction in these patients that also require attention. For these reasons, we aimed to develop criteria more specific for HS diagnosis in children with autonomic dysregulation. Symptoms of autonomic dysregulation in patients with HS can range from relatively mild (craniopharyngioma) to severe (ROHHAD). Milder symptoms are easily overlooked but can contribute to the diagnosis of HS.

To improve the sensitivity of our diagnostic score for HS, we here propose to include autonomic dysfunction as an addition to the 2023 diagnostic criteria. Using this amendment to the current diagnostic score, we evaluated four ROHHAD patients known in our clinic.

## Methods

### Development of the add-on score for autonomic dysregulation

Items for scoring autonomic dysfunction were identified by combining existing assessments (COMPASS-31 (14)) and symptoms of autonomic dysregulation in ROHHAD syndrome reported in the literature (5, 12, 13, 17) with our clinical experience of patients with ROHHAD syndrome and other forms of acquired hypothalamic dysfunction.

The domains that are described in COMPASS-31 are *orthostatic intolerance*, *vasomotor*, *secretomotor*, *gastrointestinal*, *bladder,* and *pupillomotor* (14). A recent evaluation of autonomic dysfunction in 116 ROHHAD patients divided symptoms of autonomic dysregulation into thermal dysfunction, cardiovascular manifestations, excessive sweating, strabismus, gastrointestinal disturbances, sleep disturbances, abnormal pain threshold, and cold hands and feet (13). Khaytin *et al.* described ophthalmological abnormalities to include strabismus, altered pupillary response to light, oculomotor apraxia, ptosis, and decreased tearing (12). Reviewing the first 15 cases of ROHHAD syndrome extensively described by Ize-Ludlow *et al.*, no new domains or symptoms of autonomic dysregulation were identified (5). Based on expert opinion, we also added torticollis to our score of autonomic dysfunction. Torticollis, or idiopathic cervical dystonia (ICD), is not something extensively described in the literature in relation to autonomic dysfunction or ROHHAD syndrome yet; however, recently, association with other symptoms of autonomic dysfunction was shown in 20 patients with ICD (18).

Combining symptoms reported in the literature and omitting the domains of the COMPASS-31 we deemed not relevant for our population, we identified the following six domains: *ophthalmological dysfunction*, *peripheral vaso- or secretomotor tone*, *pain threshold*, *gastrointestinal or bladder motility*, *cardiovascular manifestations,* and *torticollis* (Table 1). Since presence of circadian rhythm (sleep) difficulties and temperature dysregulation were already scored in the original diagnostic criteria for HS (3), these are not part of the add-on score for autonomic dysregulation as well. We integrated the add-on score for autonomic dysregulation by categorizing the score (ranging from 0; not present to 1; mild/yes and 2; severe) and adding this as a separate item to the hypothalamic score (Table 2).

**Table 1:** Suggested add-on score: autonomic dysregulation.

Clinical symptoms*	Score
Ophthalmological dysfunction	0 = no
	1 = presence of strabismus, oculomotor apraxia, variably altered pupil reactions to light, ptosis, or altered vision (not caused by suprasellar tumor)
	2 = presence of two or more symptoms of ophthalmological dysfunction (other than altered vision)
Altered peripheral vaso- or secretomotor tone	0 = no
	1 = presence of ice-cold (swollen) hands/feet, frequent reddening of cheeks/facial flushing, aberrant sweating, dry mouth and/or dry eyes
	2 = presence of two or more symptoms of altered peripheral vaso- or secretomotor tone
Altered pain threshold (decreased or increased)	0 = no
	1 = mild; experiences little differences compared to peers
	2 = severe; e.g., no pain during venipuncture, intolerance for tight clothing or shoes
Gastrointestinal or bladder dysmotility	0 = no or mild obstipation/diarrhea
	1 = presence of constipation or diarrhea for >3 months (needing medication), and/or difficulty passing urine, completely emptying bladder, loss of bladder control
Cardiovascular manifestations	0 = no
	1 = presence of altered blood pressure regulation, orthostatic intolerance, decreased heart rate variability, arrhythmia, exercise intolerance^†^
	2 = presence of two or more symptoms
Torticollis	0 = no
	1 = presence of asymmetrical head or neck position (may be more apparent when tired)

Total score:

0–3 = no autonomic dysregulation.

4–6 = mild autonomic dysregulation.

7–10 = severe autonomic dysregulation.

* Symptoms are scored based on reporting or home measurement by parents, caregivers, or patients, and/or observations by the clinician.

^†^ Altered blood pressure regulation: hypotension, hypertension, fluctuating tension (not including hypertension due to obesity). Orthostatic intolerance: symptoms of orthostatic intolerance or objectified (by tilt test, for example). Decreased heart rate variability is defined as < −2 SDS (or <5th percentile) for age, gender, and measuring method (long-term (24 h) or short-term (2–15 min)) (23, 24, 25, 26). Arrhythmia includes irregular rhythm, bradycardia or tachycardia. Exercise intolerance is inadequate increase of heart rate during exercise or impaired recovery after exercise (27).

**Table 2:** Suggested updated criteria for diagnosis of hypothalamic syndrome.

	Score	Total score
**Clinical criteria**		
Hyperphagia	0 = no	1 = minor criterion
	1 = mild (can be controlled by parental restriction or patient itself)	2 = major criterion
	2 = mild after specific intervention for hyperphagia OR severe (cannot be controlled by parents or patient itself or steals food)	
Hypophagia/failure to thrive (diencephalic syndrome)	0 = no	1 = minor criterion
	1 = mild (can be stimulated to eat by parents/caregivers)	2 = major criterion
	2 = severe (cannot be stimulated to eat by parents/caregivers or requires tube feeding for eating)	
Body mass index (kg/m^2^)	0 = normal weight or overweight	1 = minor criterion
	1 = rapid weight gain defined as ΔBMI > +0.75 SD in the first 3 months post neuro-surgery OR ΔBMI > +1 SD in 12 months (19)	2 = major criterion
	2 = normal weight after specific intervention for hypothalamic obesity OR overweight after specific intervention for hypothalamic obesity ** OR obesity	
	(BMI using the Cole criteria* or age and gender specific)	
Pituitary dysfunction	0 = no pituitary dysfunction	1 = minor criterion
	1 = partial or complete pituitary dysfunction (with or without AVP deficiency (with adequate thirst feeling) or SiADH) OR history of central precocious puberty	2 = major criterion
	2 = (partial or complete) pituitary dysfunction including AVP deficiency AND adipsia, (inadequate thirst feeling)	
Behavioral problems	0 = none	1 = minor criterion
	1 = mild (can be corrected by parents/caregivers) OR score >22.2 on PWSBQ	2 = major criterion
	2 = mild after specific intervention for hypothalamic behavioral problems OR severe (cannot be corrected by parents/caregivers, requires specialist treatment) OR score >55.7 on PWSBQ	
Sleep disorder	0 = no, Epworth scale score 0–10	1 = minor criterion
	1 = mild (one or more sleep symptoms without disruption of school and/or family) OR Epworth scale score 11–17	2 = major criterion
	2 = mild after specific intervention such as melatonin OR severe (disrupts school and/or family, diagnosis of obstructive sleep apnea syndrome OR Epworth scale score >18)	
Core temperature regulation disorder	0 = no	1 = minor criterion
	1 = mild, core-temperature multiple times between 35 and 36 degrees or above 37.5	2 = major criterion
	2 = severe (needs intervention such as specialized heat clothing), core-temperature measured below <35 degrees, OR temperature spikes >38 degrees	
Autonomic dysregulation	0 = no, score 0–3	1 = minor criterion
	1 = mild, score 4–6	2 = major criterion
	2 = severe, score 7–10	
**Pre-** **test** ** probability**		
For children with a suprasellar tumor	0 = grade 0: no hypothalamic involvement or lesion	1 = minor criterion
Radiological (MRI) criteria: Müller grading	1 = grade I: hypothalamic involvement or lesion of the anterior hypothalamus that does not involve the hypothalamic area of the mammillary bodies and beyond	2 = major criterion
	2 = grade II: hypothalamic involvement or lesion of the anterior and posterior hypothalamic area	
For children with no suprasellar tumor	0 = no known genetic syndrome or diagnosis associated with hypothalamic dysfunction	1 = minor criterion
Genetic syndrome or associated diagnosis present?	1 = genetic syndrome or diagnosis present which may be associated with hypothalamic dysfunction (e.g., Smith–Magenis syndrome)	2 = major criterion
	2 = genetic syndrome or diagnosis present of which has been proven to be associated with hypothalamic dysfunction, such as Prader-will syndrome OR central hypoventilation (without central hypoventilation syndrome) OR hypothalamic inflammation on MRI OR neuro-endocrine tumor (NET)	

Data from van Santen *et al.* (3).

* Overweight and obesity in infants 0–2 years is defined according to the international cut-off points of the World Health Organization using BMI > 2.0 SDS and BMI > 3.0 respectively. Overweight and obesity for children aged ≥ 2 years is defined according to the international cut-off points of Cole *et al.*, using the international cut off points of body mass index by sex and age for overweight and obesity;

** Specific intervention for hypothalamic obesity includes treatment with dextro-amphetamine, caffeine and ephedrine-HCl, mazindol, methylphenidate, octreotide, diazoxide and metformin, beloranib, exenatide, liraglutide tesomet, oxytocin and naltrexone, carbetocin or bariatic surgery.

Hypothalamic syndrome (HS) may be considered present in case of:

- ≥ 3 major criteria OR.

- ‘At least’ four minor criteria OR.

- ‘At least’ one minor radiological and two major OR.

- Two major and ‘at least’ two minor OR.

- One major radiological and major obesity OR.

- One major radiological criterion and ‘at least’ two minor criteria.

#### Update of the current diagnostic criteria for the presence of hypothalamic syndrome, combined with autonomic dysregulation add-on

While adding autonomic dysregulation to the original hypothalamic diagnostic criteria, we also critically re-assessed the existing criteria to form an updated version (Table 2). Due to the fact that not all children with HS have obesity yet but may also, especially post-operatively, have significant weight gain, we changed the scoring of BMI by adding BMI increase to the diagnostic score (19). Second, we updated the item on temperature dysregulation. We renamed the item to ‘core temperature regulation disorder’ in order to distinguish this item from the peripheral vasomotor dysregulation related to dysautonomia, resulting in ice-cold extremities. We also added temperature spikes (an abrupt temperature >38 degrees Celsius with no infection) as a scoring item since this may be a sign of hypothalamic temperature dysregulation as well.

#### Calculation of total score

The total score for autonomic dysregulation was categorized as no/mild/severe and added as a minor (mild autonomic dysregulation) or major (severe autonomic dysregulation) criterion to the diagnostic criteria for HS. A score of 0–3 was considered no autonomic dysregulation, a score of 4–6 mild, and 7–10 severe autonomic dysregulation.

### Feasibility of the updated diagnostic criteria

To assess the feasibility and possible added value of our proposed amendment and changes to the diagnostic criteria for HS, we performed a retrospective evaluation of four ROHHAD patients known in our clinic. Inclusion criteria for this cohort were diagnosis of ROHHAD syndrome by a pediatric endocrinologist at an age ≤18 years.

#### Data collection

Medical records were used for retrospective data collection. Patient characteristics, time of diagnosis, and duration of follow-up were reported. ROHHAD onset was defined as first presentation with symptoms related to ROHHAD syndrome at the pediatrician, as patients were in follow-up from that moment on, and there is usually a delay in the actual diagnosis.

First, hypothalamic dysfunction was scored using the diagnostic criteria by van Santen *et al.* at first presentation and at last visit of follow-up (3). All domains were scored separately, and the presence of HS according to the diagnostic criteria was scored. Symptoms in domains not reported on at first presentation were assumed to be not severe (at least). In addition, the added items on autonomic dysfunction were scored at follow-up. Since this was a retrospective evaluation, no questionnaires were used, and items were scored as reported in the medical record. If items had not been reported in the medical records, it was scored as missing. Items on autonomic dysregulation were scored as present if it was reported at any point during follow-up.

## Results

### Study population

In our endocrine clinical department, five ROHHAD patients were known. Of these five patients, one patient did not provide informed consent for use of their data. Therefore, the data of four ROHHAD patients were included in this evaluation. All four patients were female and <6 years of age at time of onset of ROHHAD symptoms, with a follow-up time of >2 years after onset of first ROHHAD symptoms (Table 3).

**Table 3:** Descriptive characteristics of included ROHHAD cohort.

Patient characteristics	*n* = 4
Sex, female, % (*n*/N)	100 (4/4)
Age at ROHHAD onset, median in years (range)*	2.5 (2.0–5.5)
Follow-up interval, median in years (range)	4.8 (2.5–23.7)
Age at last follow-up visit, median in years (range)	9.4 (4.6–25.7)
Hypothalamic obesity at presentation, % (*n*/N)	75 (3/4)
Central hypoventilation, % (*n*/N)	75 (3/4)
Neuroblastoma–ganglioneuroma present, % (*n*/N)	75 (3/4)

* Age at first signs/symptoms: rapid onset obesity, hyperphagia, autonomic dysfunction, ganglioneuroma/(ganglio)neuroblastoma.

### Scoring of ROHHAD patients using the 2023 hypothalamic syndrome diagnostic criteria

First, we assessed the patients using the original version of the diagnostic criteria for HS at first presentation and at last visit of follow-up (Table 4). Initially, one out of four patients met the 2023 criteria for HS. At last visit of follow-up, all four patients fulfilled the diagnostic criteria for HS.

**Table 4:** Assessment of hypothalamic score in four patients with ROHHAD syndrome.

Clinical criteria	Score per patient							
	#1		#2		#3		#4	
	Dx	FU	Dx	FU	Dx	FU	Dx	FU
Hyperphagia	1	1	1	1	1	2	1	2
Hypophagia	0	0	0	0	0	0	0	0
BMI	0	2	2	2	2	2	2	2
Behavior	2	2	-	1	-	0	0	1
Sleep	1	2	-	0	0	0	0	2
Temperature regulation	-	2	-	0	-	2	-	2
Pituitary function	1	2	1	1	0	2	0	1
Pre-test probability	0	2	0	2	0	2	0	2
Presence of HS (yes/no)	Yes	Yes	No	Yes	No	Yes	No	Yes

Dx = score at first presentation of ROHHAD symptoms; FU = score at last moment of follow-up. 0 = no or normal, 1 = minor criterion, 2 = major criterion. – = not assessed at first presentation. See Table 2 for the full items scored.

### Add-on score for autonomic dysregulation

Subsequently, we used the add-on score for scoring of autonomic dysregulation in the four patients (Table 5). All patients scored positive on at least three different domains of autonomic dysfunction. The median score was 9 out of 10 (range: 4–9). All patients experienced at least some form of ophthalmological dysfunction or altered peripheral vaso-/secretomotor tone. Every pre-defined domain was scored by at least 75% of the patients (3/4). Data were missing on several items. Heart rate variability was not measured in any of the patients. Finally, we integrated the score for autonomic dysregulation with the updated diagnostic criteria at the last moment of follow-up (Table 6). In 75% (3/4) of the patients, autonomic dysregulation was present as a major criterion, and all patients fulfilled the updated criteria for HS.

**Table 5:** Assessment of add-on score for autonomic dysregulation in four patients with ROHHAD syndrome.

Clinical symptoms	Score per patient			
	#1	#2	#3	#4
Ophthalmological dysfunction	2	2	2	1
Strabismus	Yes	Yes	Yes	Yes
Oculomotor apraxia	Yes	Yes	Yes	No
Altered pupil reactions to light	Yes	No	Yes	No
Ptosis	No	No	No	No
Altered vision	Yes	Yes	Yes	Yes
Altered peripheral vaso- or secretomotor tone	2	1	2	2
Ice-cold extremities	Yes	Yes	Yes	Yes
Facial flushing/redness	Yes	Yes	Yes	Yes
Aberrant sweating	Yes	No	Yes	Yes
Dry mouth	Yes	No	No	Yes
Dry eyes	No	No	Yes	Yes
Pain threshold	2	0	1	2
Elevated pain threshold	Yes	No	Yes	Yes
Decreased pain threshold	Yes	No	No	No
Gastrointestinal or bladder dysmotility	1	0	1	1
Constipation	Yes	No	Yes	Yes
Diarrhea	No	No	No	No
Bladder dysfunction	Yes	No	Yes	No
Cardiovascular manifestations	1	0	2	2
Blood pressure regulation	-	-	Yes	-
Orthostatic intolerance	No	No	Yes	Yes
Decreased HRV	-	-	-	-
Arrhythmia (bradycardia, tachycardia, irregular rhythm)	Yes	No	Yes	Yes
Exercise intolerance	No	-	Yes	Yes
Torticollis	0	1	1	1
Total (out of 10)	9	4	9	9

0 = no or normal, 1 = yes/minor, 2 = major, – = not assessed. HRV = heart rate variability.

See Table 1 for the full items. Total score:

0–3 = no autonomic dysregulation.

4–6 = mild autonomic dysregulation.

7–10 = severe autonomic dysregulation.

**Table 6:** Assessment of HS with the updated hypothalamic score at follow-up.

Clinical criteria	Score per patient			
	**#1**	**#2**	**#3**	**#4**
Hyperphagia	1	1	2	2
Hypophagia	0	0	0	0
BMI	2	2	2	2
Behavior	2	1	0	1
Sleep	2	0	0	2
Core temperature regulation	2	0	2	2
Pituitary function	2	1	2	1
Autonomic dysregulation	2	1	2	2
Pre-test probability	2	2	2	2
Presence of HS (yes/no)	Yes	Yes	Yes	Yes

0 = no or normal, 1 = minor criterion, 2 = major criterion. See Table 2 for the full items.

Hypothalamic syndrome (HS) may be considered present in case of:

- ≥ 3 major criteria OR.

- ‘At least’ four minor criteria OR.

- ‘At least’ one minor radiological and two major OR.

- Two major and ‘at least’ two minor OR.

- One major radiological and major obesity OR.

- One major radiological criterion and ‘at least’ two minor criteria.

## Discussion

Here we propose an update of the diagnostic criteria for HS previously published in 2023, including a first scoring system for the signs and symptoms of autonomic dysregulation in specific forms of HS, such as in ROHHAD syndrome. The novel scoring system proposed by van Santen *et al.* in 2023 (3) was an important first step in earlier recognition and diagnosis of HS. However, the signs and symptoms of autonomic dysfunction were not represented sufficiently in the initial scoring system. With advancing insight, we judged the original diagnostic score insufficient for early recognition of rare diseases such as ROHHAD syndrome. Comparable to the other signs of HS, signs of autonomic dysfunction are easily overlooked by clinicians and caregivers, especially with initial or mild presentation (13). With this updated and improved scoring system, we aim to improve recognition of hypothalamic dysfunction and reduce delay in diagnoses of rare diseases such as ROHHAD syndrome.

In addition to improvement of diagnosis of ROHHAD syndrome, this updated score may also prove to be useful for children with congenital or acquired forms of hypothalamic dysfunction. In addition, children with craniopharyngioma may have altered pain sensations, facial flushing, and gastrointestinal symptoms, which may not always be recognized (20). Applying this improved score in future cohorts will help to clarify to what extent autonomic dysregulation is present in other forms of hypothalamic dysfunction.

Comparable to the large amount of missing data in the first described cohort in 2023 (5), we also encountered missing data regarding signs and symptoms of autonomic regulation, implying that in current care, presence of autonomic dysfunction is not assessed well enough. This is partly caused by the unawareness of dysfunction of this domain and the lack of easily measurable parameters. We hope that the clinical characterization and perception of signs and symptoms of hypothalamic dysfunction, including autonomic dysregulation, will be improved after introduction of the score (3).

We assessed the score for feasibility in our small cohort and found that all patients displayed signs and symptoms of autonomic dysregulation in at least three different domains. Objective scoring of autonomic dysfunction is challenging. Suggested tests are, for example, pupillometry or thermoregulatory sweat tests to objectify pupillary reactions to light, sweating, and variation in core and peripheral temperature in ROHHAD syndrome (12). The Composite Autonomic Scoring Scale (CASS) is another validated test set for autonomic reflexes, focusing mostly on cardiovascular manifestations. A 10-point scoring scale was developed using a quantitative sudomotor axon reflex test and heart rate and blood pressure measurements during tilt test, deep breathing, and Valsalva maneuver (21). However, all of these tests are not done routinely, and some are not even available at all in many academic hospitals or expert centers. In addition, the correlation between subjective capturing of dysautonomia and objective measurements using, for example, CASS is questionable (22). Not all symptoms of autonomic dysregulation as seen in ROHHAD syndrome are represented in these objective measurements. Therefore, we consider our proposed scoring for autonomic dysregulation a good and clinically useful alternative, combining more subjective scoring with objective measurements where possible.

At first presentation, only 1/4 of the ROHHAD patients met the diagnostic criteria for HS published in 2023 (5). One of the patients did not present with obesity initially but did show rapid weight gain, illustrating the added value of our adjustment to the BMI criterion. At follow-up, all four patients in our cohort did meet the original criteria for HS, which was partly due to the fact that they were all already clinically diagnosed with ROHHAD syndrome. As ROHHAD syndrome is progressive, they displayed more signs and symptoms of hypothalamic dysfunction at the latest visit of follow-up than they did at time of first presentation. The score at time of diagnosis illustrates that we may overestimate the sensitivity of the original hypothalamic diagnostic criteria in ROHHAD patients. In ROHHAD syndrome, signs and symptoms develop in time. For this reason, it is even more important to develop diagnostic criteria reflecting all possible relevant symptoms of hypothalamic dysfunction, including autonomic dysregulation. By adding autonomic dysregulation as an extra domain to the diagnostic criteria, without changing the scoring system, HS will be diagnosed earlier on.

Prospective use of our score will help to recognize and systematically evaluate the smaller, more difficult-to-identify symptoms of autonomic dysregulation in children with different forms of hypothalamic dysfunction such as craniopharyngioma and Prader–Willi syndrome (14, 15). Since this is currently not evaluated structurally, it is possible that milder signs and symptoms of autonomic dysfunction are missed while they may affect quality of daily life.

In conclusion, we propose an updated score for signs and symptoms of hypothalamic dysfunction, including autonomic dysregulation. We consider our updated score feasible, although it was tested only in a small retrospective cohort. We suggest testing the score prospectively in patients suspected for ROHHAD syndrome or other causes of (acquired) hypothalamic dysfunction. Where possible, signs and symptoms can be assessed prospectively using validated questionnaires or objective measurements. We expect that this improved scoring system will be of added value to systematically score signs and symptoms of hypothalamic and autonomic dysregulation, improving diagnosis and follow-up care.

## Declaration of interest

NJD is local co-investigator of the Transcend Study (Rhythm Pharmaceuticals) and received an unrestricted grant from Rhythm Pharmaceuticals. HLM has received reimbursement of participation fees for scientific meetings and continuing medical education events from the following companies: Ferring, Pfizer, Sandoz/Hexal, Novo Nordisk, IPSEN, Merck Serono, and Rhythm Pharmaceuticals. HLM has received reimbursement of travel expenses from Merck Serono, Rhythm Pharmaceuticals, and IPSEN, and lecture honoraria from Pfizer and Rhythm Pharmaceuticals. HMvS is local primary investigator of the Transcend Study (Rhythm Pharmaceuticals), has received funding from Pfizer, Novo Nordisk, and Rhythm Pharmaceuticals for the organization of the ESPE Science Symposium as well as the 6th Craniopharyngioma Post-graduate Course, has received reimbursement of travel expenses for a scientific meeting by Rhythm Pharmaceuticals, and received funding for research projects from both Rhythm Pharmaceuticals and Pfizer.

## Funding

This work did not receive any specific grant from any funding agency in the public, commercial, or not-for-profit sector. NJD has received an unrestricted grant from Rhythm Pharmaceuticals. HLM/the KRANIOPHARYNGEOM studies have been funded by grants (HLM, DKS2014.13) of the German Childhood Cancer Foundation, Bonn, Germany.

## Author contribution statement

NJ Doelman-Oldenburger and HM van Santen were responsible for conceptualization, formal analysis, investigation, and writing the original draft. NJ Doelman-Oldenburger, HL Müller, and HM van Santen were responsible for methodology, and writing review and editing.

## Data availability

The data underlying this article cannot be shared publicly due to the privacy of individuals that participated in the study. The data will be shared on reasonable request to the corresponding author.

## Ethics

Patients and/or parents gave informed consent for use of their data. Only pseudonymized data were used in this study. The Dutch institutional review board decided that no additional procedures were required (MEC-2016-739). This research was performed in accordance with the Declaration of Helsinki.
